# Validity of the Polar M430 Activity Monitor in Free-Living Conditions: Validation Study

**DOI:** 10.2196/14438

**Published:** 2019-08-16

**Authors:** André Henriksen, Sameline Grimsgaard, Alexander Horsch, Gunnar Hartvigsen, Laila Hopstock

**Affiliations:** 1 Department of Community Medicine UiT The Arctic University of Norway Tromsø Norway; 2 Department of Computer Science UiT The Arctic University of Norway Tromsø Norway

**Keywords:** actigraphy, fitness trackers, motor activity, validation studies

## Abstract

**Background:**

Accelerometers, often in conjunction with heart rate sensors, are extensively used to track physical activity (PA) in research. Research-grade instruments are often expensive and have limited battery capacity, limited storage, and high participant burden. Consumer-based activity trackers are equipped with similar technology and designed for long-term wear, and can therefore potentially be used in research.

**Objective:**

We aimed to assess the criterion validity of the Polar M430 sport watch, compared with 2 research-grade instruments (ActiGraph and Actiheart), worn on 4 different locations using 1- and 3-axis accelerometers.

**Methods:**

A total of 50 participants wore 2 ActiGraphs (wrist and hip), 2 Actihearts (upper and lower chest position), and 1 Polar M430 sport watch for 1 full day. We compared reported time (minutes) spent in sedentary behavior and in light, moderate, vigorous, and moderate to vigorous PA, step counts, activity energy expenditure, and total energy expenditure between devices. We used Pearson correlations, intraclass correlations, mean absolute percentage errors (MAPEs), and Bland-Altman plots to assess criterion validity.

**Results:**

Pearson correlations between the Polar M430 and all research-grade instruments were moderate or stronger for vigorous PA (*r* range .59-.76), moderate to vigorous PA (*r* range .51-.75), steps (*r* range .85-.87), total energy expenditure (*r* range .88-.94), and activity energy expenditure (*r* range .74-.79). Bland-Altman plots showed higher agreement for higher intensities of PA. MAPE was high for most outcomes. Only total energy expenditure measured by the hip-worn ActiGraph and both Actiheart positions had acceptable or close to acceptable errors with MAPEs of 6.94% (ActiGraph, 3 axes), 8.26% (ActiGraph, 1 axis), 14.54% (Actiheart, upper position), and 14.37% (Actiheart, lower position). The wrist-worn ActiGraph had a MAPE of 15.94% for measuring steps. All other outcomes had a MAPE of 22% or higher. For most outcomes, the Polar M430 was most strongly correlated with the hip-worn triaxial ActiGraph, with a moderate or strong Pearson correlation for sedentary behavior (*r*=.52) and for light (*r*=.7), moderate (*r*=.57), vigorous (*r*=.76), and moderate to vigorous (*r*=.75) PA. In addition, correlations were strong or very strong for activity energy expenditure (*r*=.75), steps (*r*=.85), and total energy expenditure (*r*=.91).

**Conclusions:**

The Polar M430 can potentially be used as an addition to established research-grade instruments to collect some PA variables over a prolonged period. However, due to the high MAPE of most outcomes, only total energy expenditure can be trusted to provide close to valid results. Depending on the variable, the Polar M430 over- or underreported most metrics, and may therefore be better suited to report changes in PA over time for some outcomes, rather than as an accurate instrument for PA status in a population.

## Introduction

### Background

Lack of physical activity (PA) is the fourth-leading risk factor for global mortality, and the World Health Organization recommends at least 150 minutes weekly of moderate-intensity PA (eg, 30 minutes of moderate PA, 5 times per week) or 75 minutes weekly of vigorous-intensity PA for adults, and 60 minutes weekly of moderate to vigorous PA (MVPA) for children and adolescents [1]. However, worldwide, these recommendations are not reached by 80% of adolescents and 31% of adults (ranging from 17% in Southeast Asia to 43% in the eastern Mediterranean and the Americas) [2]. Two national reports from the Norwegian Directorate of Health show that, in the Norwegian population, these recommendation were reached by only 20% in 2009 [3] and 32% in 2015 [4].

Accelerometers and combined sensing (ie, accelerometers and heart rate) are used to track PA. Research-grade instruments are often expensive and have limited battery capacity, limited storage, and high participant burden. Consumer-based activity trackers, on the other hand, are designed for long-term wear, equipped with similar technologies, generally cheaper, and less intrusive, and can potentially track PA for research purposes.

Consumer-based activity trackers are increasingly being evaluated for use in research. Recent examples includes Lawrie et al [5] and Beukenhorst et al [6], who included smart watches in their research protocols. The major limitation of these devices is the limited knowledge of device validity. Due to the rapid growth of new devices, high-quality validation studies of emerging models are needed [7]. Specifically, to our knowledge, no validation study on the Polar M430 has been conducted to date. Most previous validation studies have compared multiple consumer devices with 1- or 2-criterion instruments (eg, [8,9]). In this study, we compared 1 consumer device with multiple criteria, placed on multiple locations, and analyzing 1 and 3 axes of the accelerometer.

### Objective

The aim of this study was to assess the criterion validity of time (in minutes) spent in various PA intensity zones, step counts, and energy expenditure (EE) between the Polar M430 and 2 extensively used research-grade instruments (ActiGraph and Actiheart) worn on 4 different locations using uniaxial and triaxial measurements in free-living conditions. We used multiple criteria because we wanted to show how the choice of criterion and placement affects outcomes. The ActiGraph can be considered a reference standard for PA intensity in free-living people, but because the Actiheart also has a heart rate sensor, it can be an attractive alternative in many cases.

## Methods

### Sample

We recruited 50 participants, who were eligible for inclusion if they were 18 years of age or older with normal physical function. We used convenience sampling to maximize ranges for weight, height, body mass index, age, and sex.

### Instruments

The Polar M430 (Polar Electro Oy, Kempele, Finland), released in 2017, is a sport watch with a 6–light-emitting diode wrist-based optical heart rate sensor and a 50-Hz triaxial accelerometer for tracking PA. It weighs 51 g, with 20 days of battery life.

ActiGraph wGT3X-BT (ActiGraph LLC, Pensacola, FL, USA) is a 19-g triaxial accelerometer with a 30- to 100-Hz sampling rate, to be worn on the wrist, hip, ankle, or thigh, with 25 days of battery life. ActiGraph has been previously validated for sedentary behavior [10-12], PA intensity for both uniaxial [12] and triaxial [13] acceleration, step counting [14], and EE [15].

The Actiheart (CamNtech Ltd, Cambridge, UK) is a 10-g uniaxial accelerometer with 32-Hz sampling rate and additional electrocardiography with 128-Hz sampling rate, to be worn on the chest, with 21 days of battery life. The Actiheart is extensively used to measure EE, and has been shown by Brage et al to produce valid estimations for EE both in laboratory settings [16] and under free-living conditions [17].

### Procedure

**Figure 1:**
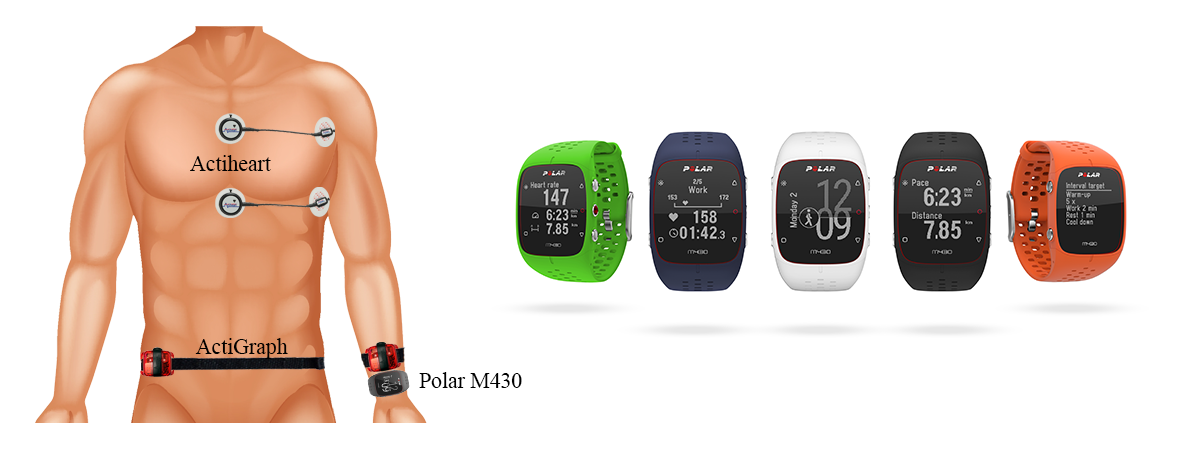
Instrument placement and Polar M430 illustrations.

We used self-reported information on height, weight, age, sex, and dominant hand to initialize the devices. The Polar M430 and an ActiGraph (attached with an elastic band) were placed on the wrist of the nondominant hand. One ActiGraph was placed on the right hip (attached with an elastic band). One Actiheart was placed approximately at the level of the second intercostal space at the sternum (medial part) and to the left (lateral part). The second Actiheart was placed approximately at the level of the fifth intercostal space at the sternum (medial part) and to the left (lateral part). The Actihearts were attached with 2 Red Dot 2238 electrodes (3M, St Paul, MN, USA) each. Table 1 [18] gives the setup used for all instruments and Figure 1 shows the placement of each instrument.

Devices were attached by 1 of 2 researchers after agreement of method in accordance with manufacturer recommendations. Participants were instructed to wear all instruments at all times except for temporarily removing the ActiGraph for showering and water activities. Participants wore all instruments for 1 full day (24 hours). We collected data in May 2018. Participants received written and oral instructions on how to wear the devices. All participants signed an informed consent form.

**Table 1:** Device setup and output variables.

Variables		Instrument		
		ActiGraph	Actiheart	Polar M430
**Hardware and setup**				
	Epoch length (lowest available)	10 s	15 s	24 h
	Accelerometer sample rate	100 Hz	32 Hz	50 Hz
	Wear location	Nondominant wrist, right hip	Chest (V_2_), chest (V_5_)	Nondominant wrist
	Parameters	Height, weight, sex, age, wear location	Height, weight, sex, age	Height, weight, sex, age, wear location
	Software for setup and download	ActiLife 6.13.3	Actiheart 4.0.122	Polar Flow [18]
	Software for analysis	QCAT^a^/ActiLife	QCAT/Actiheart	Polar Flow
	Device model	wGT3X-BT	4	2P
	Device firmware version	1.9.2	H90.65	1.1.34
**Output variables**				
	Sitting or sedentary behavior	Yes	Yes	Yes
	Light physical activity	Yes	Yes	Yes
	Moderate physical activity	Yes	Yes	Yes
	Vigorous physical activity	Yes	Yes	Yes
	Activity energy expenditure	Yes	Yes	No
	Total energy expenditure	No	Yes	Yes
	Steps	Yes	No	Yes

^a^QCAT: Quality Control and Analysis Tool.

### Variable Creation

Using the proprietary software of ActiGraph and the Actiheart, we exported activity counts into comma-separated values files, using the lowest possible epoch setting, that is, 10- (ActiGraph) and 15- (Actiheart) second epochs. From the ActiGraph, triaxial (vertical, horizontal, lateral) counts and steps per epoch were exported. From the Actiheart, uniaxial (vertical) counts were exported. We extracted precalculated variables from the Polar M430 from Polar Flow directly. Table 1 details the software and epochs.

Due to no agreed-upon cut points for calculating PA intensity from the wrist-worn ActiGraph in adults, we applied a conversion function provided by ActiLife version 6.13.3 (after ActiLife export; ActiGraph) to the wrist-worn ActiGraph data before further analysis (Multimedia Appendix 1).

Exported comma-separated values files with epoch data were imported into the custom-made Quality Control and Analysis Tool (QCAT) developed at UiT The Arctic University of Norway and Technical University of Munich. We converted activity counts into 60-second epochs before doing further analysis. We used counts per minute (CPM) to calculate minutes in the various PA intensity zones, using several algorithms. By using QCAT, data from ActiGraph and Actiheart were analyzed by the same program and comparable variables were created. We included only valid days, a priori defined as all instruments worn at least 10 hours per day [19], in the analysis. We identified nonwear time using the triaxial wear-time algorithm of Hecht et al [20]. Multimedia Appendix 2 shows correlations between QCAT and ActiLife.

For the ActiGraph data (wrist and hip), we calculated 5 PA intensity zones using cut points defined by Freedson et al [12] and Matthews et al [21], using only the vertical axis. In addition, we used a combination of the methods of Sasaki et al [13], Kozey-Keadle et al [10], and Peterson et al [11] to generate the same PA intensity zones using all 3 axes, or vector magnitude (VM). To our knowledge, there are no agreed-upon cut points for chest-based PA counts in adults using an Actiheart. However, we used cut points identified in a study by Schrack et al [22]. We combined minutes spent in vigorous and very vigorous intensity into 1 variable. Table 2 gives an overview of each cut point set.

As QCAT does not support EE calculation, we calculated this variable from the proprietary software tools ActiLife and Actiheart. We calculated EE from ActiLife using the Freedson combination 1998 formula (Freedson et al [12] plus Williams work-energy equation) for uniaxial calculation and the Freedson VM3 combination 2011 formula (Sasaki et al [13] plus Williams work-energy equation) for triaxial calculation. We analyzed nonwear time using the default Troiano [23] settings. Actiheart uses a branched model where recorded activity and heart rate from the electrocardiogram are used together to improve EE calculations [24].

While the Actiheart reports resting EE (REE), activity EE (AEE), diet-induced thermogenesis, and total EE (TEE), the ActiGraph reports only AEE, and the Polar M430 reports only TEE. Since Actiheart used the Schofield equation [25] when calculating REE, we used the same equation to convert between AEE and TEE for the Polar M430 and the Actiheart. Furthermore, we subtracted or added, respectively, 10% of TEE to account for diet-induced thermogenesis.

The Polar M430 exports data for TEE, steps, and 5 PA intensity zones: minutes in (1) rest, (2) sitting, (3) low-intensity PA, (4) medium-intensity PA, and (5) high-intensity PA. We did not know the algorithm used by Polar to assign PA in 1 of these 5 categories, but we used the following conversion between the Polar M430 and other instruments: sitting = sedentary, low = light, medium = moderate, and high = vigorous + very vigorous PA. We did not use “minutes in rest” from the Polar M430. We compared steps only between the Polar M430 and the 2 ActiGraph locations, as this variable is not available in the exported Actiheart data. We did not compare heart rate outcomes in this analysis, as our aim was to investigate PA measures. We will address heart rate measures in a separate analysis.

**Table 2:** Alternative cut-point sets for physical activity intensity zones.

Intensity zone	ActiGraph uniaxial CPM^a^	ActiGraph triaxial CPM vector magnitude	Actiheart CPM
Sedentary	≤99	≤149	≤10
Light	100-1951	150-2689	11-95
Moderate	1952-5724	2690-6166	96-234
Vigorous	5725-9498	6167-9642	≥235
Very vigorous	≥9499	≥9643	N/A^b^

^a^CPM: counts per minute.

^b^N/A: not applicable.

### Statistical Analysis

We investigated Polar M430 validity for the following variables: sedentary behavior minutes per day, light PA minutes per day, moderate PA minutes per day, vigorous PA minutes per day, MVPA minutes per day, steps per day, AEE per day, and TEE per day. We used the Shapiro-Wilk test to test normality. We calculated and compared Pearson and Spearman correlations, with and without bootstrapping. Finally, we used the Pearson correlation coefficient, with bootstrapping, to assess the association between all instrument outcomes.

We used correlation cutoffs suggested by Evans [26]: very weak, less than .2; weak, .2-.4; moderate, .4-.6; strong, .6-.8; and very strong, greater than .8. We also calculated the intraclass correlation coefficient (ICC) to test agreement between instruments (absolute agreement, 2-way random, and single measures), which is not indicated by Pearson. We used the 95% confidence intervals of the ICC estimate to indicate poor (<.5), moderate (.5-.75), good (.75-.9), and excellent (>.9) agreement [27]. Mean absolute percentage error (MAPE) was used to calculate measurement error between devices for each outcome. There is no agreed-upon cutoff for MAPE, but previous validation studies have used a MAPE of less than 5% [9] or 10% [28,29] to indicate low error.

We also used Bland-Altman plots to assess the agreement between instrument outcomes [30]. Bland-Altman limits of agreement (LoA) indicate the mean difference between 2 instruments, when comparing the mean for each outcome. A positive mean value indicates an overreporting from the Polar M430. The width of the upper and lower LoA indicates the agreement between instruments, where a narrower range indicates a higher agreement.

For each variable, we present (as a figure or multimedia appendix) a scatterplot and a Bland-Altman plot for each criterion. In the scatterplot, the blue straight line shows the fit line for the Pearson correlations. The black dashed line shows how a perfect correlation and agreement would appear, and can be used, together with the ICC, to see how much the Polar M430 over- or underreported the variables. In the Bland-Altman plot, the blue line indicates the mean difference between the Polar M430 and each criterion. Red lines show the upper and lower LoA.

Finally, we performed sensitivity and specificity tests to evaluate the ability of the Polar M430 to identify a target of 10,000 steps/day [31]. We did not report sensitivity and specificity for the recommended 30 minutes of MVPA per day, because the Polar M430 recorded at least 30 minutes of MVPA for all participants. All statistical analysis were performed using R version 3.5.3 (R Foundation).

### Ethics Approval and Consent to Participate

The Norwegian regional committees for medical and health research ethics reviewed the study (2019/557/REK nord). All participants gave informed and written consent. This study was conducted in accordance with the 1964 Declaration of Helsinki and its later amendments.

## Results

### Participant Demographics and Wear Time

Table 3 presents participants’ height, weight, body mass index, age, and sex.

All ActiGraphs had a wear time of at least 10 hours and were included in the analysis. Recording on 1 Actiheart in the upper position failed, and we excluded it from the analysis. Two Actihearts were incorrectly initialized and were excluded from the TEE and AEE analyses. Although 7 Actihearts in the upper position and 5 Actihearts in the lower position had less than 10 hours of wear time, we did not exclude these because the participants informed us that they did not remove the device and manual review of the activity data indicated misclassification of nonwear and sleep.

### Polar M430 Validity and Agreement

Multimedia Appendix 3 shows all outcomes for all criteria. Table 4 gives an overview of group data for all variables from the Polar M430. The tables in Multimedia Appendix 4 present all outcomes and group variables for each variable and all criteria.

**Table 3:** Participant characteristics (N=50).

Variable	Value	Range
Height (cm), mean (SD)	173.7 (10.1)	152-193
Weight (kg), mean (SD)	75.3 (16.4)	49-125
Body mass index (kg/m^2^), mean (SD)	24.7 (3.6)	19.0-33.6
Age (years), mean (SD)	45.1 (15.5)	19-74
Females, n (%)	24 (48)	N/A^a^

^a^N/A: not applicable.

**Table 4:** Data of exported variables from the Polar M430 (N=50).

Variable	Value
Sedentary behavior (minutes), mean (SD)	500.61 (110.78)
Light physical activity (minutes), mean (SD)	308.45 (96.40)
Moderate physical activity (minutes), mean (SD)	98.10 (48.71)
Vigorous physical activity (minutes), mean (SD)	25.55 (37.27)
Moderate to vigorous physical activity (minutes), mean (SD)	123.65 (67.50)
Total energy expenditure (kcal), mean (SD)	2591.5 (619.1)
Activity energy expenditure (kcal), mean (SD)	N/A^a^
Steps, n (%)	13,426 (4775)

^a^N/A: not applicable.

### Sedentary Behavior

Only the hip-worn ActiGraph VM gave a moderate Pearson correlation with the Polar M430. The remaining criteria gave a weak or very weak correlation. All ICC agreements were poor. The Bland-Altman LoA indicated underreporting of sedentary behavior by the Polar M430 compared with the hip-worn ActiGraph, and overreporting of the remaining criteria. All MAPEs were high. Table A in Multimedia Appendix 4 provides details of all criteria. Multimedia Appendix 5 gives correlations and Bland-Altman plots for the Polar M430 against each criterion.

### Light Physical Activity

The hip-worn ActiGraph and both Actihearts gave a strong Pearson correlation with the Polar M430. The highest ICC agreement was shown for the hip-worn ActiGraph CPM, with a poor to moderate ICC. The Bland-Altman LoA indicated an overreporting of light PA by the Polar M430 compared with the hip-worn ActiGraph CPM and both Actihearts, and an underreporting for the remaining criteria. All MAPEs were high. Table B in Multimedia Appendix 4 provides details of all criteria. Multimedia Appendix 6 gives correlations and Bland-Altman plots for the Polar M430 against each criterion.

### Moderate Physical Activity

All criteria except the Actiheart in the upper position gave a moderate Pearson correlation with the Polar M430. The highest ICC agreement was shown for the Actiheart in the lower position, with a poor to moderate ICC. The Bland-Altman LoA indicated an overreporting of moderate PA by the Polar M430 compared with the hip-worn ActiGraph CPM and both Actihearts, and an underreporting for the remaining criteria. All MAPEs were high. Table C in Multimedia Appendix 4 provides details of all criteria. Multimedia Appendix 7 gives correlations and Bland-Altman plots for the Polar M430 against each criterion.

### Vigorous Physical Activity

The hip-worn ActiGraph gave a strong Pearson correlation with the Polar M430. The wrist-worn ActiGraph reported 0 minutes in vigorous PA and were therefore excluded from analysis. The Actiheart in the upper and lower position gave a strong and moderate correlation, respectively. The highest ICC agreement was shown for the hip-worn ActiGraph VM, with a poor to good ICC. The Bland-Altman LoA indicated an overreporting of vigorous PA by the Polar M430 compared with the hip-worn ActiGraph, and an underreporting for both Actihearts. All MAPEs were high. Table D in Multimedia Appendix 4 provides details of all criteria. Multimedia Appendix 8 gives correlations and Bland-Altman plots for the Polar M430 against each criterion.

### Moderate to Vigorous Physical Activity

**Figure 2:**
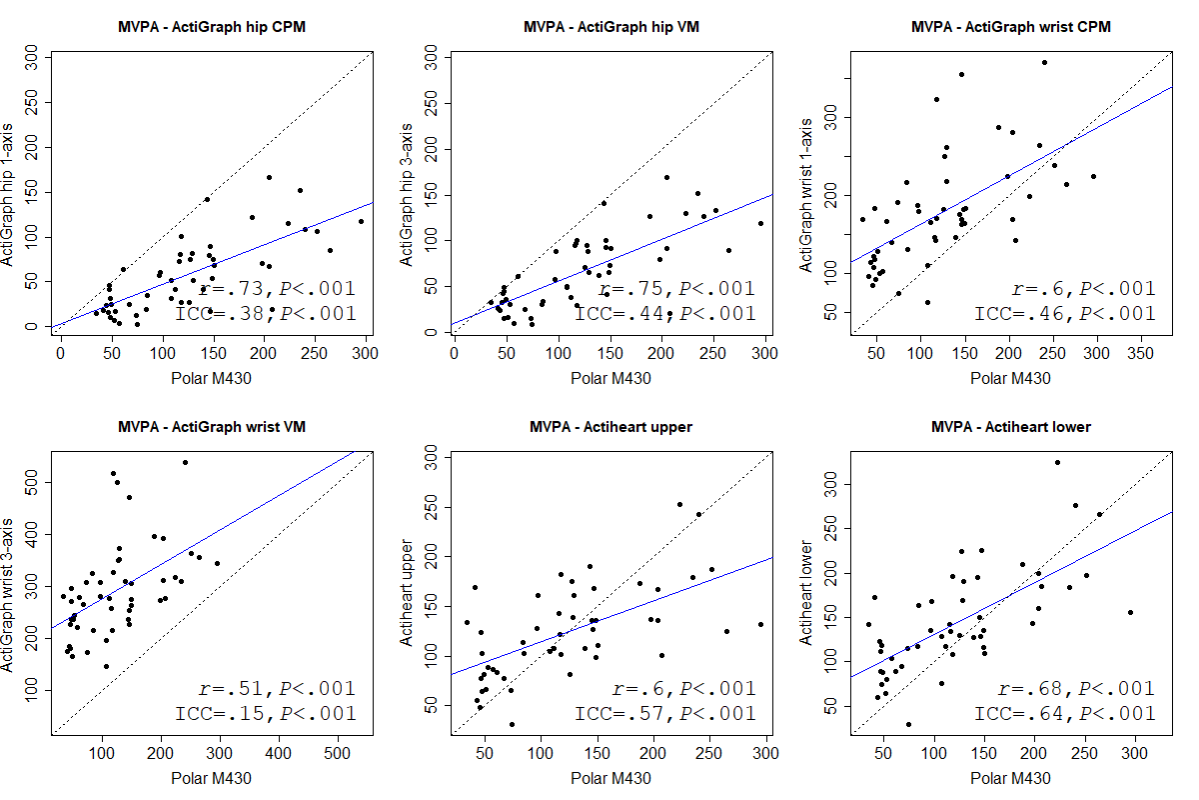
Correlation between the Polar M430 and all criterion measures for moderate to vigorous physical activity (MVPA). CPM: counts per minute; ICC: intraclass correlation coefficient; VM: vector magnitude.

**Figure 3:**
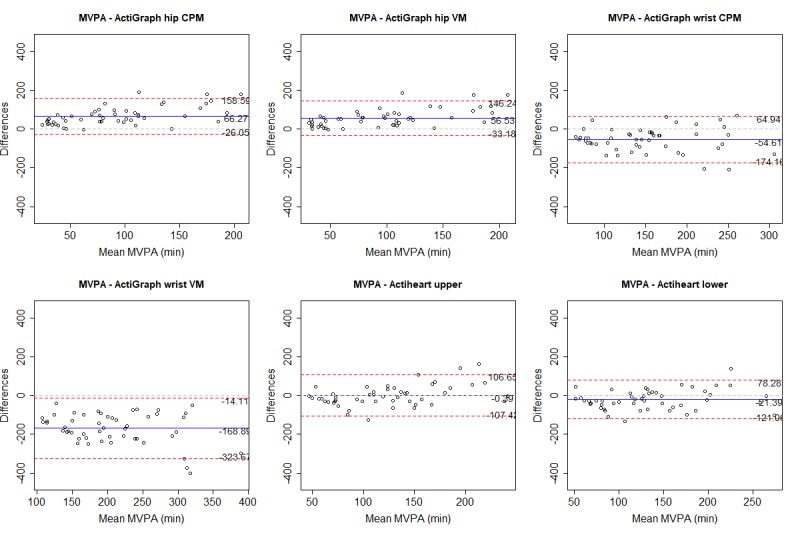
Bland-Altman plots for the Polar M430 and each criterion measure for moderate to vigorous physical activity (MVPA). Numbers are mean difference and upper and lower limits of agreement (95% CI). CPM: counts per minute; VM: vector magnitude.

All criteria, regardless of cut points and number of axes considered, gave a moderately or strongly significant Pearson correlation when comparing MVPA for the Polar M430. The hip-worn ActiGraph VM had the strongest correlation. The highest ICC agreement was shown for the Actiheart in the lower position, with a poor to good ICC. The Bland-Altman LoA indicated an overreporting of MVPA by the Polar M430 compared with the hip-worn ActiGraph, a minor underreporting for the Actiheart in the upper position, and an underreporting for the wrist-worn ActiGraph and the Actiheart in the lower position. All MAPEs were high. Table E in Multimedia Appendix 4 provides details of all criteria. Figures 2 and 3 show correlations and Bland-Altman plots, respectively, for the Polar M430 against each criterion.

### Activity Energy Expenditure

All criteria showed a strong and significant Pearson correlation for AEE, where the wrist-worn ActiGraph VM was marginally stronger than the other criteria. ICC agreement was highest for the hip-worn ActiGraph VM with a moderate to good agreement. The Bland-Altman LoA showed an overreporting of AEE by the Polar M430 compared with the hip-worn ActiGraph and an underreporting for the wrist-worn ActiGraph and both Actihearts. All MAPEs were high. Table F in Multimedia Appendix 4 provides details of all criteria. Multimedia Appendix 9 gives correlations and Bland-Altman plots for the Polar M430 against each criterion. Multimedia Appendix 10 gives a combined plot for AEE and TEE.

### Total Energy Expenditure

**Figure 4:**
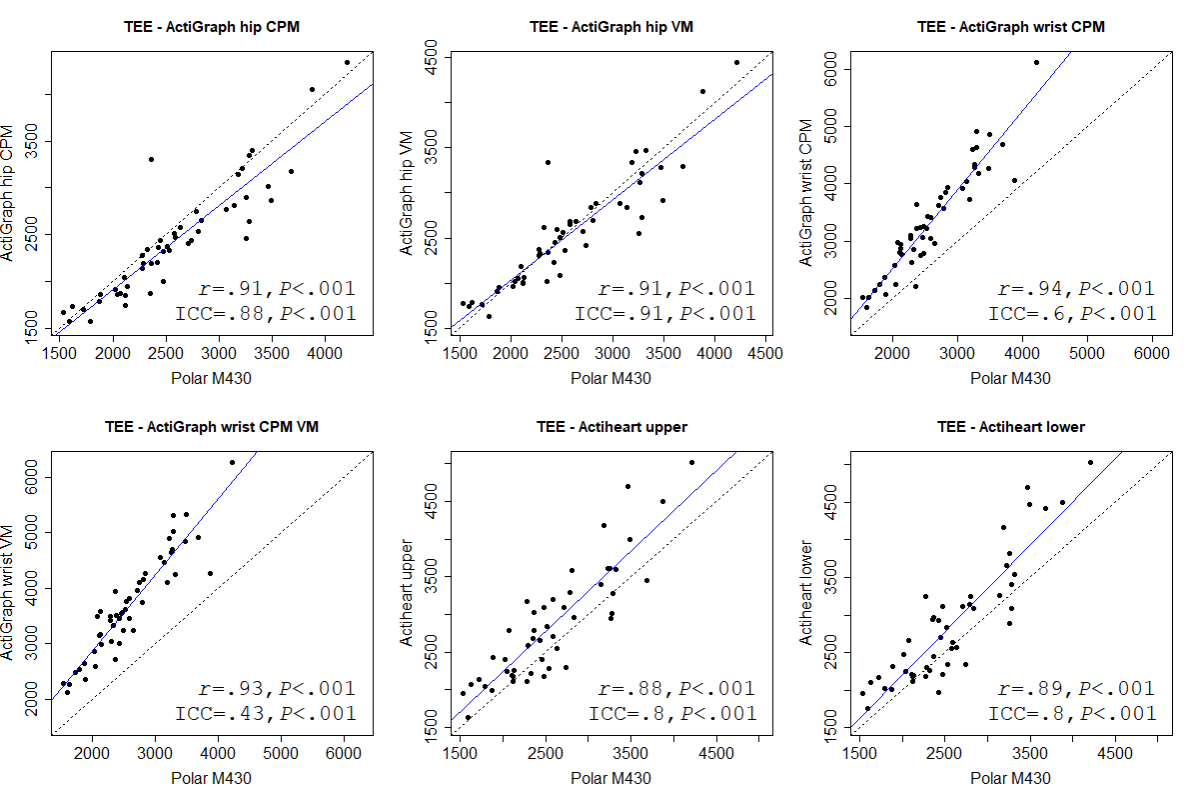
Correlation between the Polar M430 and each criterion measure for total energy expenditure (TEE). CPM: counts per minute; ICC: intraclass correlation coefficient; VM: vector magnitude.

**Figure 5:**
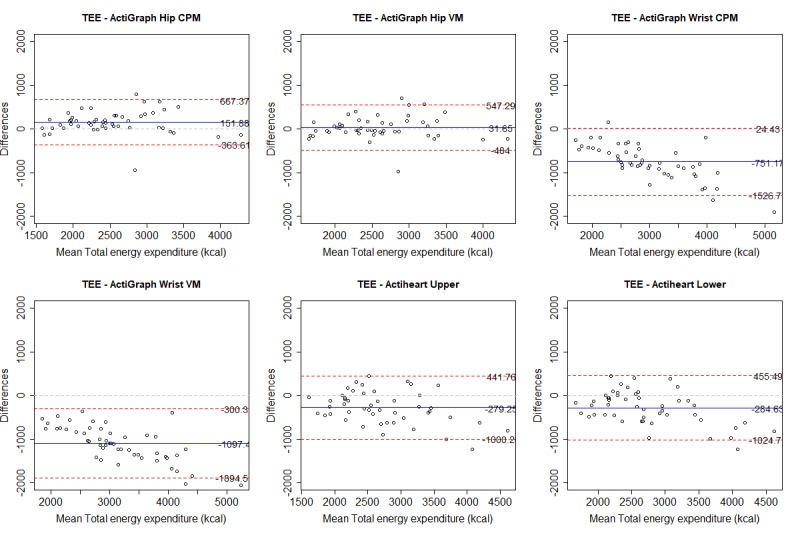
Bland-Altman plots for the Polar M430 and each criterion measure for total energy expenditure (TEE). Numbers are mean difference and upper and lower limits of agreement (95% CI). CPM: counts per minute; VM: vector magnitude.

All criteria showed a very strong and significant Pearson correlation for TEE. The correlation for wrist-worn ActiGraph CPM was marginally stronger than other ActiGraphs. ICC agreement was highest for the hip-worn ActiGraph VM, with good to excellent agreement. The Bland-Altman LoA showed an overreporting of TEE by the Polar M430 compared with the hip-worn ActiGraph, and an underreporting for remaining criteria. The hip-worn ActiGraph had an acceptable MAPE of 6.94% (VM) and 8.26% (CPM). the remaining criteria had a high MAPE. Table G in Multimedia Appendix 4 provides details of all criteria. ActiGraph does not report TEE, and group data are therefore not available. Figures 4 and 5 show correlations and Bland-Altman plots, respectively, for the Polar M430 against each criterion.

### Steps

**Figure 6:**
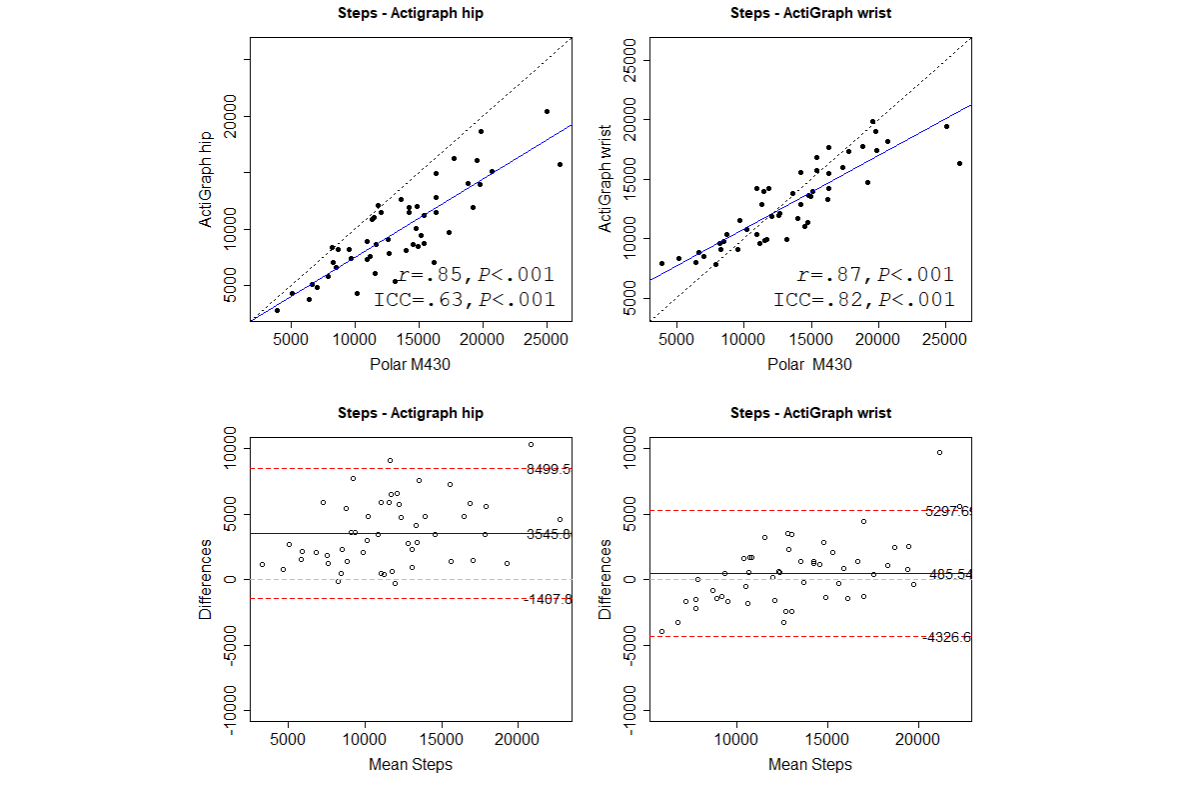
Correlations and Bland-Altman plots for the Polar M430 and the wrist-worn and hip-worn ActiGraph for steps. Numbers in the Bland-Altman plots are mean difference and upper and lower limits of agreement (95% CI). ICC: intraclass correlation coefficient.

There was a very strong significant, and approximately equal, correlation between the Polar M430 and both the wrist-worn and hip-worn ActiGraph when measuring steps. ICC agreement was moderate to good for both locations. The Bland-Altman plot showed that the Polar M430 overreported steps for both placements of the ActiGraph, but at a higher rate on the hip-worn ActiGraph. Both MAPEs were high, but the hip-worn ActiGraph had the lowest MAPE. Table H in Multimedia Appendix 4 provides details of all criteria. Figure 6 shows correlations and Bland-Altman plots for the Polar M430 against both criteria.

Sensitivity (true-positive) analysis showed that the Polar M430, compared with the hip-worn ActiGraph, identified all cases in which a participant achieved 10,000 steps/day. For the wrist-worn ActiGraph, sensitivity was .94. Specificity, the ability of the Polar M430 to correctly identify those not achieving the 10,000 step/day target was .43 for the hip-worn ActiGraph and .71 for the wrist-worn ActiGraph.

## Discussion

### Principal Findings

We have shown how the available variables correlate and agree between the Polar M430 and 6 different combinations of device, placement, and number of accelerometer axes. For most outcomes, the Polar M430 showed the strongest correlation with the hip-worn triaxial ActiGraph (VM). Similarly, agreement was most often highest (or almost as high) when we compared the Polar M430 with this criterion. Exceptions are MVPA and moderate PA, where the Actiheart in the lower position showed a somewhat higher agreement.

A previous study by Tudor-Locke et al [32] showed that the hip-worn ActiGraph had a higher accuracy for step counting than the wrist-worn ActiGraph in laboratory settings. Under free-living conditions, the same study showed that the ActiGraph detected more steps when placed on the wrist. It is therefore possible to conclude that, although our study showed that the wrist-worn ActiGraph had a higher correlation, higher agreement, and lower MAPE, the true step counts may be closer to the numbers reported by the hip-worn ActiGraph. Similarly, studies comparing how wear location affected PA intensity [33] and EE [34,35] outcomes showed that the hip-worn ActiGraph is more accurate than the wrist-worn ActiGraph.

When compared with the hip-worn ActiGraph VM, the Polar M430 had a very strong correlation for TEE and steps, a strong correlation for AEE, MVPA, light PA, and vigorous PA, and a moderate correlation for sedentary behavior and moderate PA. Bland-Altman plots showed that the mean agreement was higher for higher intensities of PA, with underreporting by the Polar M430 for sedentary behavior and light PA, and overreporting for the remaining variables. Sensitivity analysis also indicated that the Polar M430 overreported the number of steps. However, MAPE was high for most variables, and only TEE had an acceptable MAPE of 6.9%. Multimedia Appendix 11 and Multimedia Appendix 12 give correlations and Bland-Altman plots, respectively, for the Polar M430 and the hip-worn ActiGraph VM for all variables.

MVPA was strongly correlated for all criteria except 1 (ie, wrist-worn ActiGraph VM), and all criteria gave a strong correlation for AEE and a very strong correlation for TEE and steps. For the hip-worn ActiGraph, most outcomes showed a stronger correlation when using the triaxial variable than the uniaxial variable. For all criteria, all correlations for TEE were stronger and all MAPEs were smaller than for AEE. This is expected, as REE constitutes between 60% and 75% of TEE [36]. Except for sedentary behavior and moderate PA, outcomes were similar for the upper and lower position of the Actiheart. This is in accordance with a previous study by Brage et al [37], in which position did not affect outcome significantly.

### Comparison With Previous Studies

We identified 12 previous studies that compared wrist-worn Polar devices with an objective criterion measure for measuring steps, PA intensity, and EE. These studies tested 5 different Polar models: the Polar Loop (released in 2013), Polar V800 (released in 2014), Polar A300 (released in 2015), Polar A360 (released in 2015), and Polar M600 (released in 2016). We found no studies on the Polar M430 (released in 2017). The validity of EE, steps, and PA intensity levels for the Polar devices in these studies varied, and correlations ranged from weak to strong, depending on the study setting (laboratory vs free-living), device, and criterion measure.

We found 3 previous Polar validation studies on EE in laboratory settings showed a very weak to weak Pearson correlation for the Polar Loop (*r* range .02-.3) [38] and Polar A360 (*r*=.28) [39], and a very strong correlation for the Polar V800 (*r* range .63-.85) [28]. In free-living study participants, the Polar Loop [40], Polar A300 [41], and Polar V800 [42], showed a very strong (*r*=.9), strong (*r*=.83), and weak to moderate (*r* range .34-.69) correlation, respectively, for EE.

We found 3 studies on PA intensity levels in free-living populations showed poor agreement for the Polar A300 (ICC=.36) [41], strong to very strong Pearson correlation for the Polar V800 (*r* range .84-.93) [42], and moderate Spearman correlation and poor agreement for MVPA on the Polar M600 (ρ=.53, ICC=.38) [43]. We found no studies comparing PA intensity levels conducted in laboratory settings.

A total of 5 studies compared steps in laboratory settings. The Polar Loop was tested in 4 studies, where Wahl et al (*r* range .06-.83) [38], Wang et al (correlation not given) [44], and Fokkema et al (*r* range .08-.26) [9], showed low validity for steps, with a higher validity for higher walking speeds in 1 study (Wahl et al [38]). An et al [45], on the other hand, found higher validity for this device (*r* range .4-.7). Bunn et al [46] tested the Polar A360 and also found it to have low validity (r range –.24 to .49). In addition, 4 studies compared steps in free-living populations. The Polar Loop showed a strong to very strong Pearson correlation (*r* range .7-.89) [47], the Polar A300 showed a very strong correlation (*r*=.99) [41], the Polar V800 showed a very strong correlation (*r* range .89-.92) [42]), and the Polar M600 showed good agreement (ICC=.7) and a strong Spearman correlation (ρ=.85) [43].

The results from previous studies showed that the validity of Polar devices, ranging from the Polar Loop, released in 2013, to the Polar M600, released in 2016, was highly dependent on the study setting. Studies conducted in free-living populations seem to agree that EE was reasonably valid, but not always. Our study also showed a strong correlation for AEE and a very strong correlation for TEE, for some criteria. The correlations for MVPA were stronger in our study than in all other studies. Results from previous research on step counting in free-living populations showed similar strong correlations to those found in our study.

With the exception of the Polar Loop, there are a limited number of studies for each device. For all other devices, only 1 or 2 studies were available for a given device, and at most 1 per device in free-living populations. In addition, previous studies used a range of criteria, and as we found in our study, correlations between the Polar M430 and each criterion can be very different depending on which criterion is used for comparison. It is therefore difficult to compare our results with previous validation studies. However, because all of the previous validation studies were conducted on older devices, it is reasonable that our results showed stronger correlations and higher agreements, as modern devices are likely to be more accurate than older devices.

Other studies on non-Polar consumer-based activity trackers generally agreed that the validity of step was high, but validity for EE was lower. In a 2015 systematic review, Evenson et al [48] concluded that, for consumer-based activity trackers such as Jawbone and Fitbit, validity of steps was high, but validity for EE was lower. Similarly, Feehan et al [49] systematically reviewed Fitbit devices and found that validity for EE was low, but validity for measuring steps was higher. Bunn et al [50] systematically reviewed validation studies testing devices by Fitbit, Garmin, Apple, Misfit, Samsung Gear, TomTom, and Lumo, and found a tendency for devices to underestimate EE and steps, but step validity was higher at higher intensities. This is partly in contrast to our study. Compared with step counting, TEE showed higher correlations for all ActiGraph outcomes. For AEE, on the other hand, step counting was more strongly correlated.

### Strengths and Limitations

The strengths of this study include the large sample size, with a wide range for participant weight, height, body mass index, and age. We compared the Polar M430 against multiple criterion measures, showing that the outcomes were highly dependent on instrument type and placement. Furthermore, we used 1 tool (QCAT) to convert all activity counts into PA intensity variables, thereby limiting the number of unknowns introduced when using multiple software packages.

Limitations are mainly related to uncertainties in cutoffs and conversions. We compared TEE and AEE between instruments because the Polar M430 did not report AEE and the ActiGraph did not report TEE. We used the same algorithm for adding and removing REE, but since we did not know how Polar calculates REE, we did not know the conversion’s accuracy. No agreed-upon cut points for PA intensity exist for the Actiheart or the wrist-worn ActiGraph, so the accuracy of related outcomes was also somewhat uncertain. We did not individually calibrate Actiheart devices, which could have given a more accurate EE measure. Finally, the Hecht 2009 nonwear time algorithm was not created for uniaxial accelerometer CPM. This likely caused misclassification between nonwear time and sedentary behavior, and lower correlation for this outcome.

### Conclusion

This first validation study of the Polar M430 indicated higher validity for MVPA, steps, and EE than with previous Polar devices. The Polar M430 can potentially be used as an addition to established research-grade instruments to collect some PA variables over a prolonged period. Depending on the variable, the Polar M430 over- or underreported most metrics and may therefore be better suited to report changes in PA over time for some outcomes, rather than as an accurate instrument for PA status in a population. Due to the high MAPE of most outcomes, only TEE or activity tracking in large samples can be trusted to provide close to valid results. Before using any consumer activity tracker or smart watch in research, we suggest piloting the selected device in the population under study. In a future study, we will attempt to create a function for converting Polar M430 reported steps, MVPA, and EE into the ActiGraph hip-worn VM equivalent, in order to determine whether such an approach can be used to better track PA status in a population over time.

## Appendix

Multimedia Appendix 1 ActiGraph wrist-to-hip activity-count conversion table.

Multimedia Appendix 2 Correlations between ActiLife and the Quality Control and Analysis Tool.

Multimedia Appendix 3 Group data and outcomes for all variables.

Multimedia Appendix 4 Tables (A to H) of group data for each criterion compared with the Polar M430 for all outcomes.

Multimedia Appendix 5 Correlations and Bland-Altman plots for the Polar M430 and each criterion measure for sedentary behavior. Numbers in the Bland-Altman plots are mean difference and upper and lower limits of agreement (95% CI).

Multimedia Appendix 6 Correlations and Bland-Altman plots for the Polar M430 and each criterion measure for light physical activity. Numbers in the Bland-Altman plots are mean difference and upper and lower limits of agreement (95% CI).

Multimedia Appendix 7 Correlations and Bland-Altman plots for the Polar M430 and each criterion measure for moderate physical activity. Numbers in the Bland-Altman plots are mean difference and upper and lower limits of agreement (95% CI).

Multimedia Appendix 8 Correlations and Bland-Altman plots for the Polar M430 and each criterion measure for vigorous physical activity. Numbers in the Bland-Altman plots are mean difference and upper and lower limits of agreement (95% CI).

Multimedia Appendix 9 Correlations and Bland-Altman plots for the Polar M430 and each criterion measure for activity energy expenditure. Numbers in the Bland-Altman plots are mean difference and upper and lower limits of agreement (95% CI).

Multimedia Appendix 10 Combined scatterplots for energy expenditure with Pearson correlations and intraclass correlations for activity energy expenditure and total energy expenditure.

Multimedia Appendix 11 Correlations for the Polar M430 and hip-worn triaxial ActiGraph for all variables.

Multimedia Appendix 12 Bland-Altman plots for the Polar M430 and hip-worn triaxial ActiGraph, for all variables. Numbers are mean difference and upper and lower limits of agreement (95% CI).

## Abbreviations

AEE: activity energy expenditure
CPM: counts per minute
EE: energy expenditure
ICC: intraclass correlation coefficient
LoA: limits of agreement
MAPE: mean absolute percentage error
MVPA: moderate to vigorous physical activity
PA: physical activity
QCAT: Quality Control and Analysis Tool
REE: resting energy expenditure
TEE: total energy expenditure
VM: vector magnitude
