# Supplementary material for: Validity of the Polar M430 Activity Monitor in Free-Living Conditions: Validation Study
Source: JMIR Form Res. 2019 Aug 16;3(3):e14438. doi: 10.2196/14438 (PMC6716339; doi:10.2196/14438)
Supplement: Multimedia Appendix 1 [file formative_v3i3e14438_app1.docx]

# Multimedia Appendix 1 – ActiGraph wrist to hip activity count conversion table

The table below gives an overview of the cut-points used by ActiLife to convert wrist-worn ActiGraph activity counts to the hip-worn ActiGraph equivalent.

| **Wrist CPM** | **Equivalent counts** |
| --- | --- |
| 0 | 0 |
| 0 – 644 | 0.5341614 * Wrist count |
| 645 – 1272 | 1.7133758 * Wrist count - 759.414013 |
| 1273 – 3806 | 0.3997632 * Wrist count + 911.501184 |
| 3807 - infinite | 0.0128995 * Wrist count + 2383.904505 |
