# Supplementary material for: Validity of the Polar M430 Activity Monitor in Free-Living Conditions: Validation Study
Source: JMIR Form Res. 2019 Aug 16;3(3):e14438. doi: 10.2196/14438 (PMC6716339; doi:10.2196/14438)
Supplement: Multimedia Appendix 2 [file formative_v3i3e14438_app2.docx]

# Multimedia Appendix 2 – Correlation between ActiLife and QCAT

The correlation between QCAT and ActiLife was found to be strong or very strong for all activity intensity zones, when using the sample defined in this paper, and when using the default setting for wear-time validation in ActiLife (i.e. Troiano [20]).

The table below gives the Pearson correlation for each PA variable when comparing triaxial hip-worn ActiGraph, using the Troiano wear-time algorithm in ActiLife and the Hecht 2009 wear-time algorithm in QCAT. Except for sedentary behaviour, correlations were very strong.

| **Variable** | **Pearson’s r** | **Lower CI** | **Upper CI** | **Significance level** |
| --- | --- | --- | --- | --- |
| Steps | 1.000 | 1.000 | 1.000 | *P* < .001 |
| Sedentary | .606 | .394 | .757 | *P* < .001 |
| Light | .977 | .959 | .987 | *P* < .001 |
| Moderate | .935 | .887 | .963 | *P* < .001 |
| Vigorous | .993 | .988 | .996 | *P* < .001 |
| MVPA | .957 | .924 | .975 | *P* < .001 |

CI: Confidence interval (95%)
