# Supplementary material for: Validity of the Polar M430 Activity Monitor in Free-Living Conditions: Validation Study
Source: JMIR Form Res. 2019 Aug 16;3(3):e14438. doi: 10.2196/14438 (PMC6716339; doi:10.2196/14438)
Supplement: Multimedia Appendix 4 [file formative_v3i3e14438_app4.docx]

**Table A.** Group data for each criterion compared with the Polar M430: output for sedentary behavior and each criterion measure.

| Measure | ActiGraph | | | | Actiheart | |
| --- | --- | --- | --- | --- | --- | --- |
|  | Hip counts per minute | Hip vector magnitude | Wrist counts per minute | Wrist vector magnitude | Upper chest | Lower chest |
|  | | | | | | |
| Number | 50 | 50 | 50 | 50 | 49 | 50 |
| Minutes in activity, mean (SD) | 738 (105) | 620 (109) | 477 (114) | 399 (106) | 398 (110) | 416 (126) |
| Pearson *r* (95% CI) | .49 (.1 to .7)^a^ | .52 (.15 to .73)^a^ | .09 (–.16 to .32) | .06 (–.21 to .3) | .05 (–.19 to .29) | .3 (.02 to .57)^b^ |
| Intraclass correlation coefficient (95% CI) | .14 (.05 to .26)^a^ | .33 (.1 to .51)^a^ | .09 (0 to .31) | .04 (0 to .21) | .04 (0 to .23) | .24 (.01 to .5)^b^ |
| Mean absolute percentage error (%) | 52.68 | 29.24 | 25.11 | 28.90 | 25.74 | 22.22 |
| Mean difference | –237.23 | –119.59 | 23.19 | 101.97 | 98.05 | 84.29 |
| Upper limit of agreement | –22.99 | 91.00 | 320.66 | 394.00 | 392.14 | 359.83 |
| Lower limit of agreement | –451.47 | –330.18 | –274.28 | –190.06 | –196.04 | –191.25 |

^a^*P*≤.001.

^b^*P*≤.05.

**Table B.** Group data for each criterion compared with the Polar M430: output for light physical activity and each criterion measure.

| Measure | ActiGraph | | | | Actiheart | |
| --- | --- | --- | --- | --- | --- | --- |
|  | Hip counts per minute | Hip vector magnitude | Wrist counts per minute | Wrist vector magnitude | Upper chest | Lower chest |
|  |  |  |  |  |  |  |
| Number | 50 | 50 | 50 | 50 | 49 | 50 |
| Minutes in activity, mean (SD) | 291 (99) | 399 (111) | 536 (81) | 501 (73) | 245 (85) | 272 (87) |
| Pearson *r* (95% CI) | .62 (.46-.75)^a^ | .7 (.53-.81)^a^ | .41 (.12-.65) ^b^ | .02 (–.23 to .29) | .69 (.52-.8)^a^ | .62 (.44-.76)^a^ |
| Intraclass correlation coefficient (95% CI) | .62 (.47-.75)^a^ | .5 (.37-.65)^a^ | .1^b^ (.03-.18) | .01 (0-.08) | .55 (.4-.68)^a^ | .58 (.42-.72)^a^ |
| Mean absolute percentage error (%) | 22.75 | 38.09 | 92.40 | 83.44 | 25.41 | 24.24 |
| Mean difference | 17.11 | –90.79 | –227.93 | –192.43 | 65.52 | 36.71 |
| Upper limit of agreement | 182.99 | 69.79 | –37.85 | 42.46 | 207.03 | 193.71 |
| Lower limit of agreement | –148.77 | –251.37 | –418.01 | –427.32 | –75.99 | –120.29 |

^a^*P*≤.001.

^b^*P*≤.05.

**Table C.** Group data for each criterion compared with the Polar M430: output for moderate physical activity and each criterion measure.

| Measure | ActiGraph | | | | Actiheart | |
| --- | --- | --- | --- | --- | --- | --- |
|  | Hip counts per minute | Hip vector magnitude | Wrist counts per minute | Wrist vector magnitude | Upper chest | Lower chest |
|  |  |  |  |  |  |  |
| Number | 50 | 50 | 50 | 50 | 49 | 50 |
| Minutes in activity, mean (SD) | 49 (36) | 56 (35) | 178 (69) | 293 (88) | 57 (25) | 71 (37) |
| Pearson *r* (95% CI) | .52 (.25-.66)^a^ | .57 (.27-.70)^a^ | .53 (.33-.72)^a^ | .53 (.33-.72)^a^ | .34 (.03-.59)^b^ | .56 (.31-.74)^a^ |
| Intraclass correlation coefficient (95% CI) | .31 (.16-.45)^a^ | .36 (0.18-0.52)^a^ | .27 (.15-.42)^a^ | .1 (.06-.17)^a^ | .18 (.02-.34)^b^ | .45 (.25-.62)^a^ |
| Mean absolute percentage error (%) | 49.72 | 40.89 | 109.11 | 250.02 | 43.93 | 40.58 |
| Mean difference | 48.72 | 41.86 | –80.16 | –194.44 | 42.02 | 27.36 |
| Upper limit of agreement | 133.01 | 121.83 | 37.20 | –48.39 | 133.52 | 108.56 |
| Lower limit of agreement | –35.57 | –38.11 | –197.52 | –340.49 | –49.48 | –53.84 |

^a^*P*≤.001.

^b^*P*≤.05.

**Table D.** Group data for each criterion compared with the Polar M430: output for vigorous physical activity and each criterion measure.

| Measure | ActiGraph | | | | Actiheart | |
| --- | --- | --- | --- | --- | --- | --- |
|  | Hip counts per minute | Hip vector magnitude | Wrist counts per minute | Wrist vector magnitude | Upper chest | Lower chest |
|  |  |  |  |  |  |  |
| Number | 50 | 50 | - | - | 49 | 50 |
| Minutes in activity, mean (SD) | 8 (21) | 11 (23) | - | - | 68 (40) | 74 (40) |
| Pearson *r* (95% CI) | .6 (.25-.82)^a^ | .76 (.52-.85)^a^ | - | - | .62 (.4-.78)^a^ | .59 (.37-.75)^a^ |
| Intraclass correlation coefficient (95% CI) | .44 (.14-.83)^a^ | .62 (.42-.88)^a^ | - | - | .39 (.21-.59)^a^ | .33 (.17-.52)^a^ |
| Mean absolute percentage error (%) | 82.59 | 79.53 | - | - | 833.33 | 953.60 |
| Mean difference | 17.55 | 14.67 | - | - | –42.41 | –48.75 |
| Upper limit of agreement | 76.16 | 63.40 | - | - | 24.11 | 20.00 |
| Lower limit of agreement | –41.06 | –34.06 | - | - | –108.92 | –117.50 |

^a^*P*≤.001.

**Table E.** Group data for each criterion compared with the Polar M430: output for moderate to vigorous physical activity and each criterion measure.

| Measure | ActiGraph | | | | Actiheart | |
| --- | --- | --- | --- | --- | --- | --- |
|  | Hip counts per minute | Hip vector magnitude | Wrist counts per minute | Wrist vector magnitude | Upper chest | Lower chest |
|  |  |  |  |  |  |  |
| Number | 50 | 50 | 50 | 50 | 49 | 50 |
| Minutes in activity, mean (SD) | 57 (41) | 67 (41) | 178 (69) | 293 (88) | 124 (47) | 145 (58) |
| Pearson *r* (95% CI) | .73 (.53-.82)^a^ | .75 (.54-.84)^a^ | .6 (.44-.73)^a^ | .51 (.34-.66)^a^ | .6 (.39-.76)^a^ | .68 (.51-.79)^a^ |
| Intraclass correlation coefficient (95% CI) | .38 (.26-.5)^a^ | .44 (.31-.57)^a^ | .46 (.31-.61)^a^ | .15 (.09-.23)^a^ | .57 (.36-.74)^a^ | .64 (.48-.77)^a^ |
| Mean absolute percentage error (%) | 53.53 | 43.49 | 79.40 | 198.61 | 44.79 | 53.87 |
| Mean difference | 66.27 | 56.53 | –54.61 | –168.89 | –0.39 | –21.39 |
| Upper limit of agreement | 158.59 | 146.24 | 64.94 | –14.11 | 106.65 | 78.28 |
| Lower limit of agreement | –26.05 | –33.18 | –174.16 | –323.67 | –107.42 | –121.06 |

^a^*P*≤.001.

**Table F.** Group data for each criterion compared with the Polar M430: output for activity energy expenditure and each criterion measure.

| Measure | ActiGraph | | | | Actiheart | |
| --- | --- | --- | --- | --- | --- | --- |
|  | Hip counts per minute | Hip vector magnitude | Wrist counts per minute | Wrist vector magnitude | Upper chest | Lower chest |
|  |  |  |  |  |  |  |
| Number | 50 | 50 | 50 | 50 | 48 | 49 |
| Kcal, mean (SD) | 603 (348) | 711 (353) | 1415 (543) | 1727 (558) | 987 (488) | 991 (525) |
| Pearson *r* (95% CI) | .75 (.5-.87)^a^ | .75 (.54-.87)^a^ | .78 (.65-.86)^a^ | .79 (.63-.87)^a^ | .74 (.57-.85)^a^ | .79 (.63-.87)^a^ |
| Intraclass correlation coefficient (95% CI) | .69 (.51-.84)^a^ | .75 (.53-.87)^a^ | .31 (.24-.41)^a^ | .2 (.15-.27)^a^ | .57 (.45-.69)^a^ | .59 (.49-.69)^a^ |
| Mean absolute percentage error (%) | 27.71 | 24.01 | 103.08 | 152.65 | 54.38 | 53.38 |
| Mean difference | 136.69 | 28.48 | –676.05 | –987.66 | –252.65 | –257.58 |
| Upper limit of agreement | 600.63 | 492.57 | 21.99 | –270.27 | 396.16 | 408.60 |
| Lower limit of agreement | –327.25 | –435.60 | –1374.09 | –1705.06 | –901.46 | –923.76 |

^a^*P*≤.001.

**Table G.** Group data for each criterion compared with the Polar M430: output for total energy expenditure and each criterion measure.

| Measure | ActiGraph | | | | Actiheart | |
| --- | --- | --- | --- | --- | --- | --- |
|  | Hip counts per minute | Hip vector magnitude | Wrist counts per minute | Wrist vector magnitude | Upper chest | Lower chest |
|  |  |  |  |  |  |  |
| Number | - | - | - | - | 48 | 49 |
| Kcal, mean (SD) | - | - | - | - | 2864 (763) | 2866 (806) |
| Pearson *r* (95% CI) | .91 (.75-.95)^a^ | .91 (.78-.95)^a^ | .94 (.88-.97)^a^ | .93 (.86-.96)^a^ | .88 (.8-.93)^a^ | .89 (.81-.94)^a^ |
| Intraclass correlation coefficient (95% CI) | .88 (.78-.94)^a^ | .91 (.8-.96)^a^ | .6 (.52-.68)^a^ | .44 (.35-.53)^a^ | .8 (.71-.86)^a^ | .8 (.73-.86)^a^ |
| Mean absolute percentage error (%) | 8.26 | 6.94 | 28.71 | 42.54 | 14.54 | 14.37 |
| Mean difference | 151.88 | 31.65 | –751.17 | –1097.40 | –279.25 | –284.63 |
| Upper limit of agreement | 667.37 | 547.29 | 24.43 | –300.30 | 441.76 | 455.49 |
| Lower limit of agreement | –363.61 | –484.00 | –1526.77 | –1894.51 | –1000.26 | –1024.75 |

^a^*P*≤.001.

**Table H.** Group data for each criterion compared with the Polar M430: output for steps and both criteria reporting steps.

| Measure | ActiGraph hip CPM | ActiGraph wrist CPM |
| --- | --- | --- |
|  |  |  |
| Number | 50 | 50 |
| Step count, mean (SD) | 9880 (3913) | 12940 (3381) |
| Pearson *r* (95% CI) | .85 (.75-.91)^a^ | .87 (.79-.92)^a^ |
| Intraclass correlation coefficient (95% CI) | .63 (.49-.75)^a^ | .82 (.7-.88)^a^ |
| Mean absolute percentage error (%) | 25.98 | 15.94 |
| Mean difference | 3546 | 486 |
| Upper limit of agreement | 8500 | 5298 |
| Lower limit of agreement | –1408 | –4327 |

^a^*P*≤.001.
