# Supplementary figures and images for: Validity of the Polar M430 Activity Monitor in Free-Living Conditions: Validation Study
Source: JMIR Form Res. 2019 Aug 16;3(3):e14438. doi: 10.2196/14438 (PMC6716339; doi:10.2196/14438)

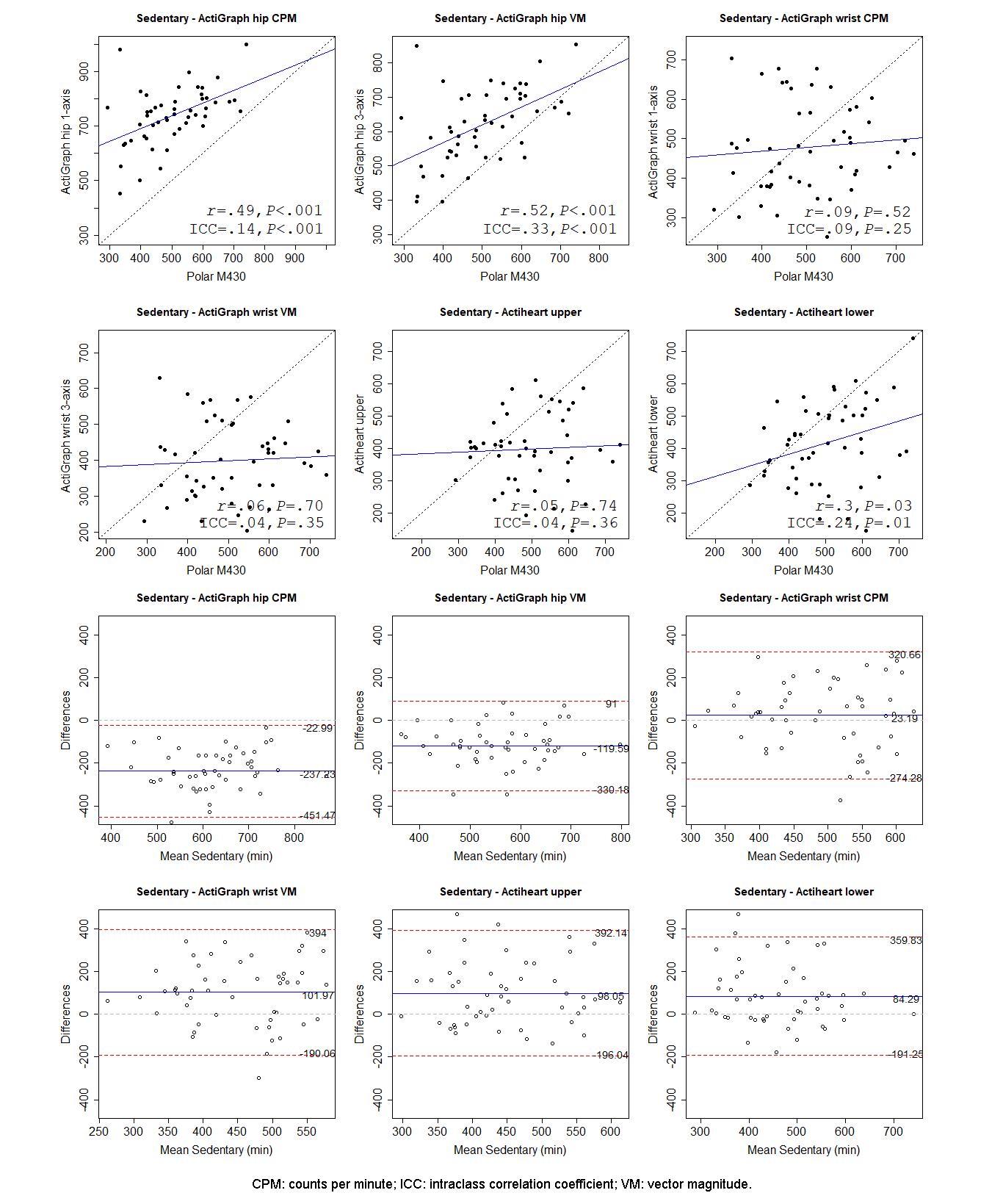

Supplement: Multimedia Appendix 5 [file formative_v3i3e14438_app5.png]

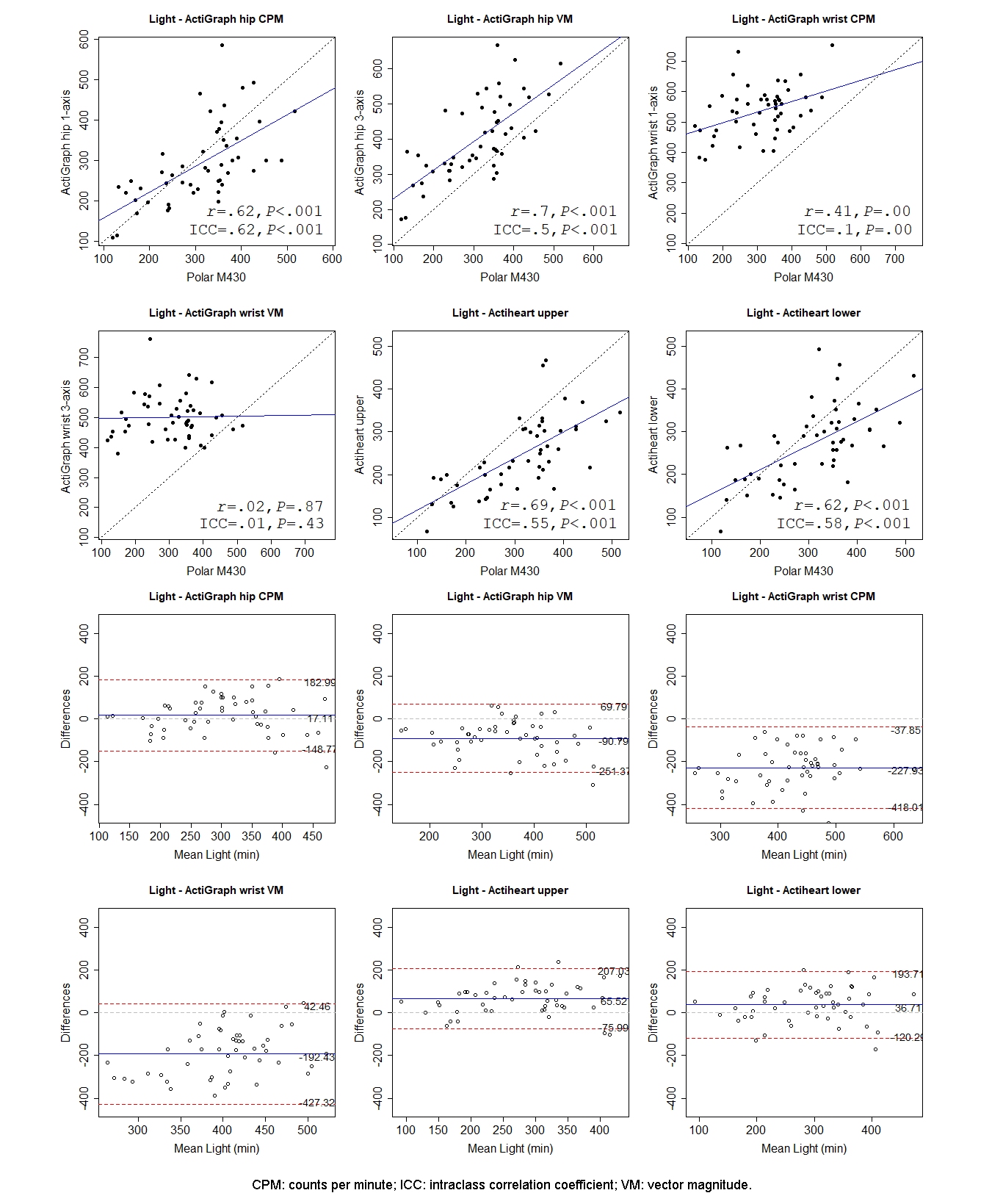

Supplement: Multimedia Appendix 6 [file formative_v3i3e14438_app6.png]

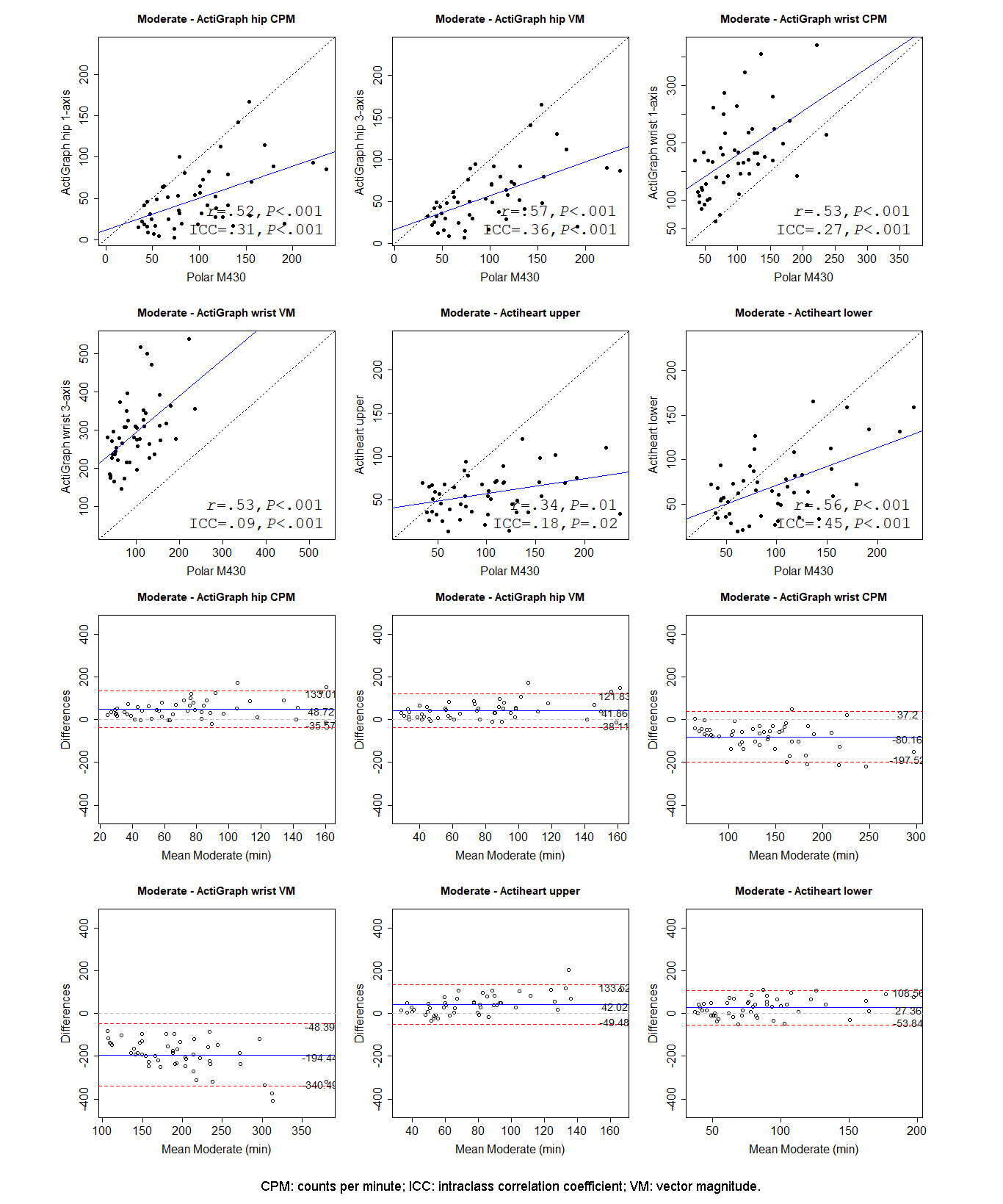

Supplement: Multimedia Appendix 7 [file formative_v3i3e14438_app7.png]

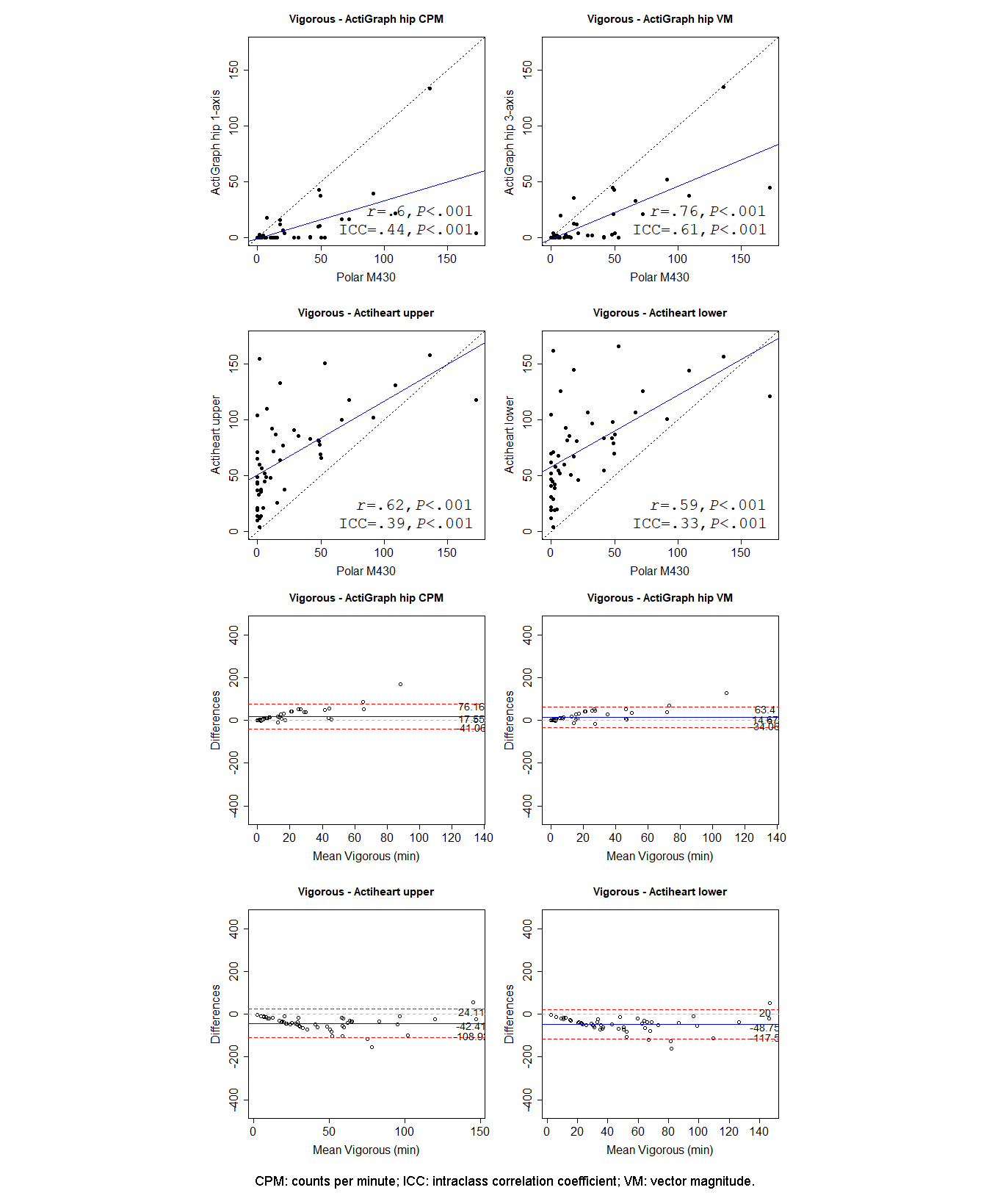

Supplement: Multimedia Appendix 8 [file formative_v3i3e14438_app8.png]

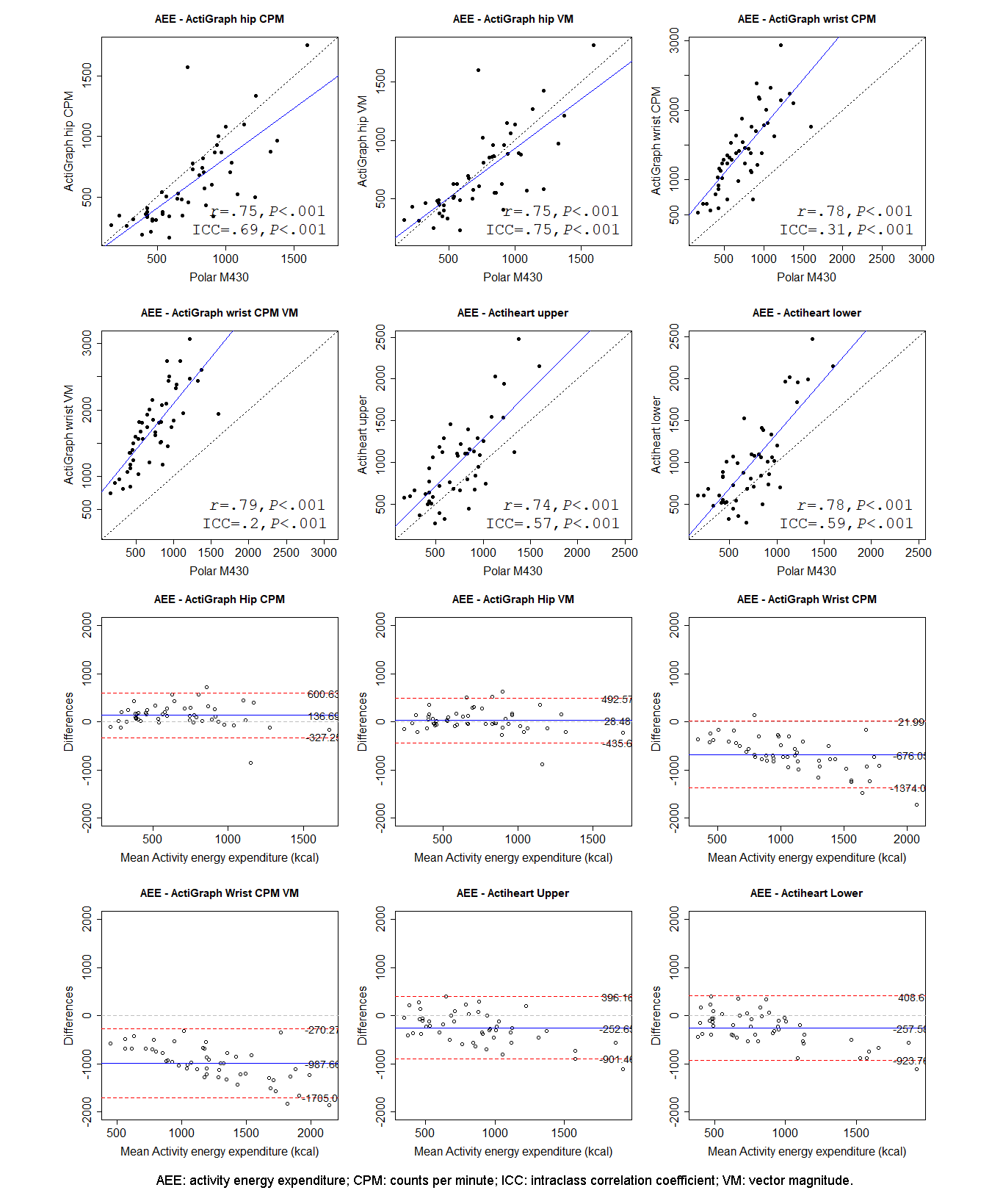

Supplement: Multimedia Appendix 9 [file formative_v3i3e14438_app9.png]

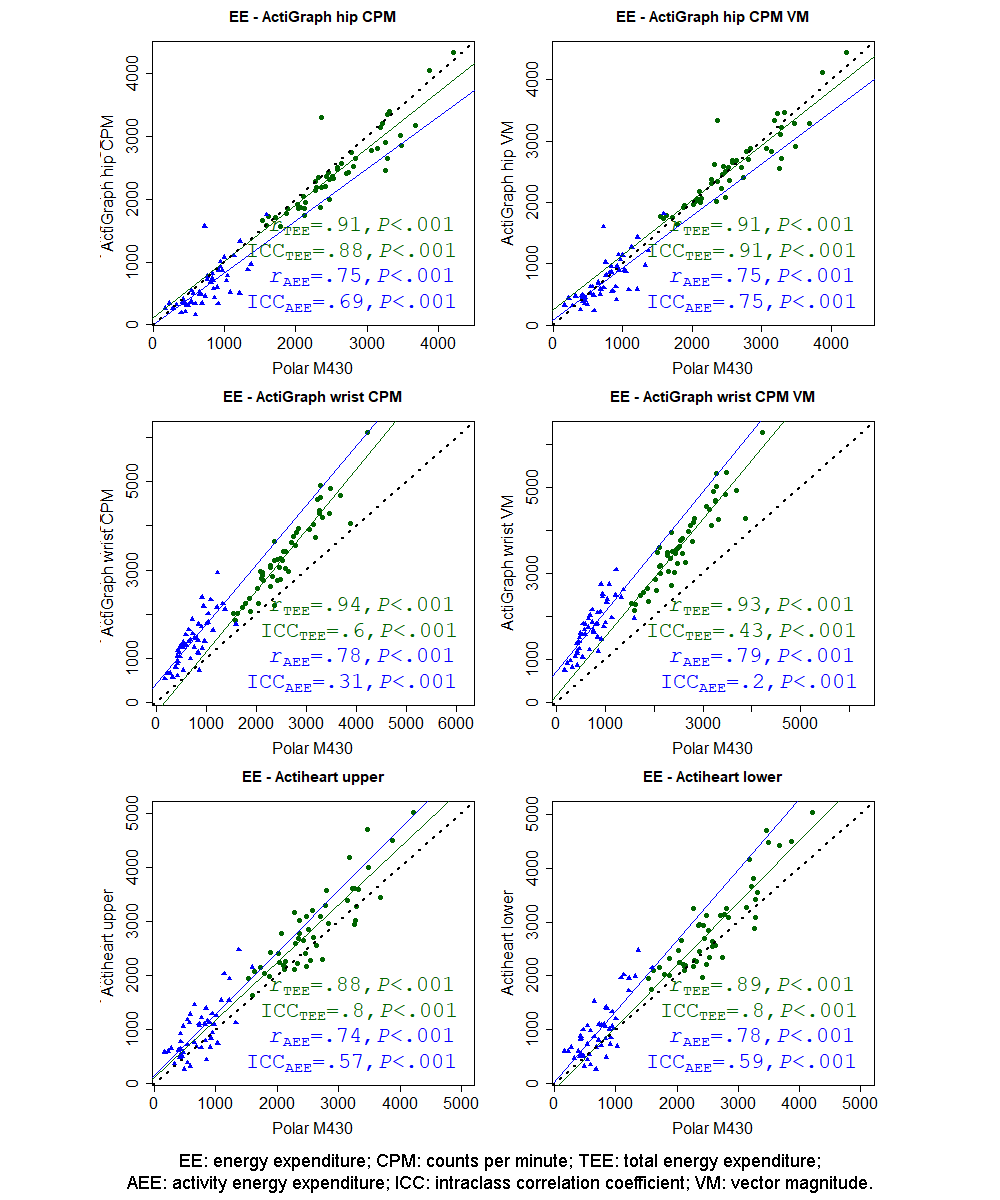

Supplement: Multimedia Appendix 10 [file formative_v3i3e14438_app10.png]

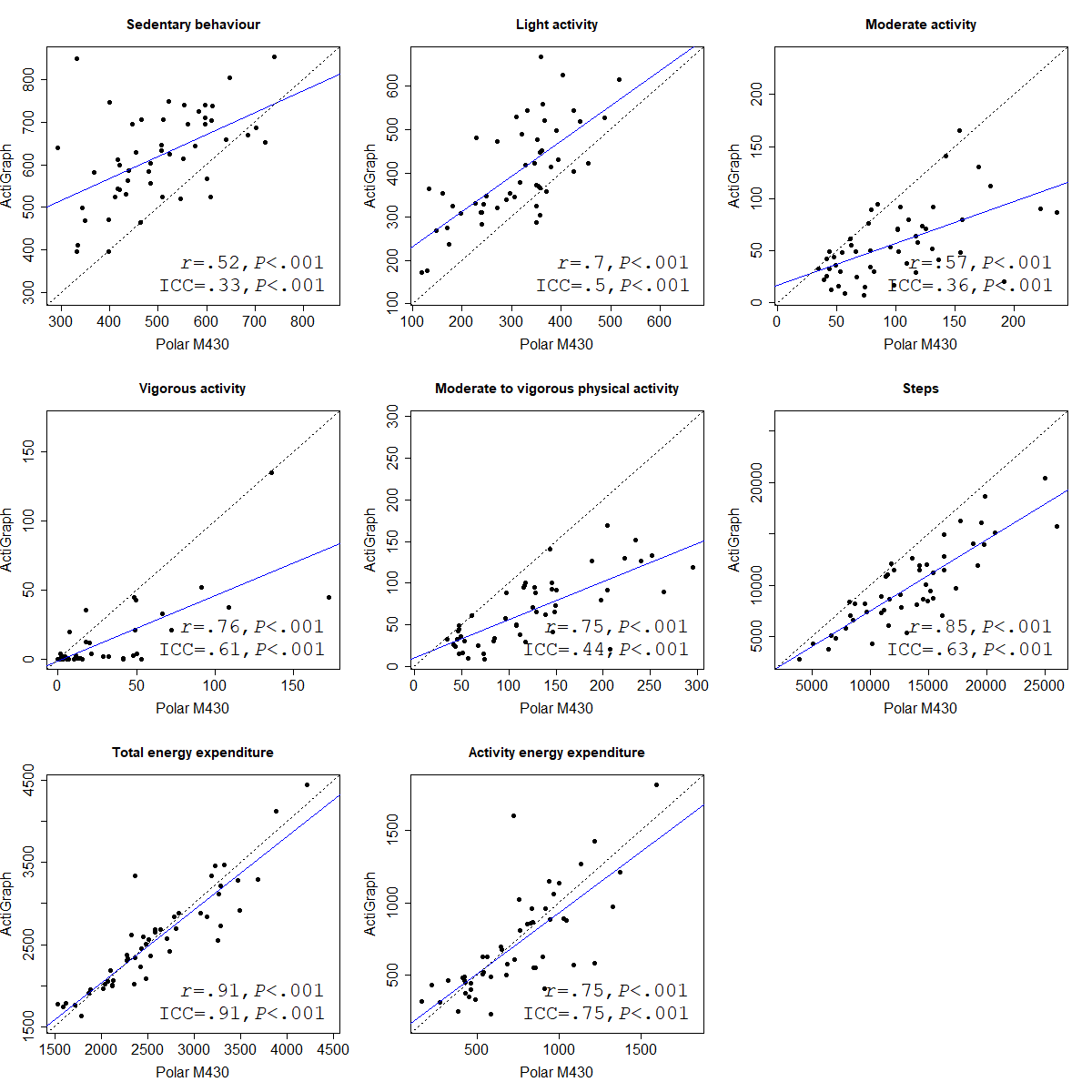

Supplement: Multimedia Appendix 11 [file formative_v3i3e14438_app11.png]

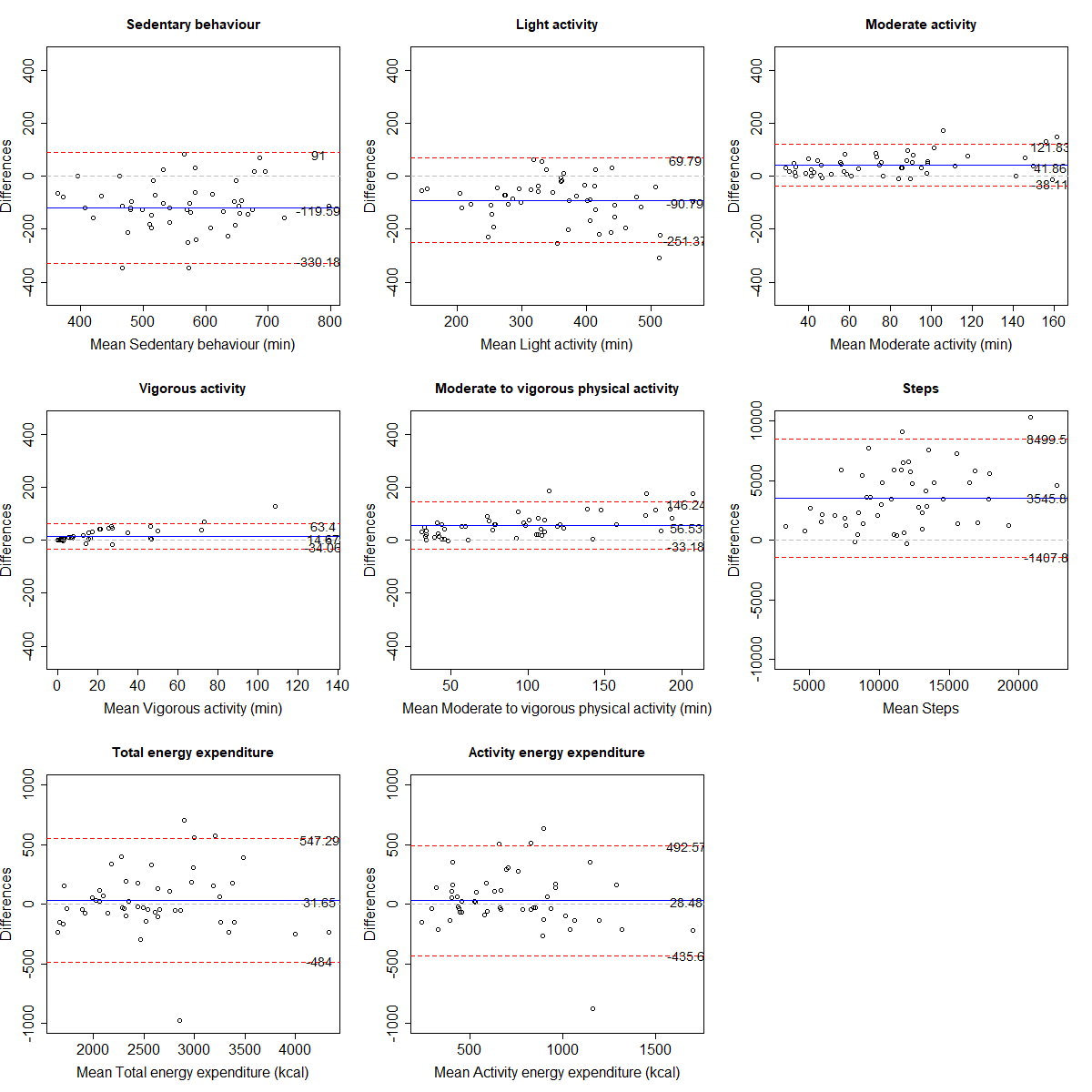

Supplement: Multimedia Appendix 12 [file formative_v3i3e14438_app12.png]
